# Association of Sociodemographic, Lifestyle, and Metabolic Risk Factors With Angiographic Severity of Coronary Artery Disease in Western Maharashtra

**DOI:** 10.7759/cureus.109339

**Published:** 2026-05-21

**Authors:** Makarand Mane, Satish R Patil, Priyanka M Mane, Sourish Hota

**Affiliations:** 1 Department of Medicine, Krishna Vishwa Vidyapeeth (Deemed to be University), Karad, IND; 2 Department of Microbiology, Krishna Vishwa Vidyapeeth (Deemed to be University), Karad, IND

**Keywords:** cad risk factors, coronary artery disease, diabetes mellitus, hypertension, india, karad, logistic regression, multi-vessel disease, rural cardiology, stemi

## Abstract

Background

Coronary artery disease (CAD) is an expanding public health problem in India, with an increasing burden in rural and peri-urban populations where patients frequently present with advanced disease. Evidence on the contribution of social and psychosocial factors to angiographic severity in these settings remains limited.

Methods

This hospital-based cross-sectional study included 100 consecutive adult patients with angiographically confirmed CAD at a tertiary care center in Western Maharashtra. Sociodemographic, lifestyle, psychosocial, and clinical data were collected using a structured proforma. Disease severity was categorized as single-vessel disease (SVD) or multi-vessel disease (MVD). Associations were assessed using Chi-square or Fisher’s exact test, followed by multivariable binary logistic regression to identify independent predictors of MVD.

Results

Multi-vessel disease was present in 61% of patients, indicating a predominance of advanced coronary involvement. The study population was largely older, with 57% aged above 60 years, and showed male predominance (61%). Most participants were from rural areas (62%) and lived with family (72%). Hypertension (59%) and diabetes (37%) were common, and a high burden of biochemical abnormalities was observed, including deranged lipid profile (80%), positive troponin-I (80%), and elevated creatine phosphokinase-MB (CPK-MB) (76%). Although trends suggested higher MVD in older age groups and across certain psychosocial categories, none of the sociodemographic, lifestyle, clinical, or biochemical variables showed a statistically significant association with disease extent. In multivariable analysis, living arrangement emerged as the only independent predictor, with individuals living alone or outside family settings having significantly lower odds of MVD (adjusted OR = 0.13; 95% CI: 0.02-0.76; p = 0.024).

Conclusion

A high burden of multi-vessel CAD at presentation reflects the substantial underlying disease load in this population. While conventional risk factors did not independently predict angiographic severity, this highlights the complexity of CAD and the potential contribution of broader determinants beyond traditional clinical profiles. The significant association observed with living arrangement emphasizes the relevance of social context in shaping disease presentation and supports the incorporation of social determinants into cardiovascular risk assessment. These findings provide a valuable foundation for future research and may help guide more comprehensive and context-sensitive approaches to risk stratification in similar populations.

## Introduction

Cardiovascular disease (CVD) is the number one cause of death globally, with about 17.9 million deaths per year, which is almost 32 percent of the total deaths in the world [1]. Coronary artery disease (CAD) is the largest contributor to morbidity and mortality among CVDs, and low- and middle-income countries (LMICs) have almost 80% of the burden in the world [2]. India, especially, is undergoing a fast-growing CAD epidemic, with earlier onset, almost 10 years before Western populations, and more severe clinical manifestations, such as multi-vessel disease, larger infarct size, and higher case-fatality rates [3,4]. India has experienced a major epidemiological change in the last few decades, where non-communicable diseases are becoming more prevalent among rural and peri-urban populations [5]. However, in contrast to previous notions that CAD was a disease of urban prosperity, new data show that it is becoming more common in rural areas, with the prevalence of hypertension, diabetes mellitus, and other cardiometabolic risk factors on the rise [5,6].

Research has also indicated an increasing number of cardiovascular risk factors among Indian groups, including poor glycemic control, dyslipidemia, and lifestyle changes such as decreased physical activity and unhealthy eating habits [7,8]. Furthermore, South Asians have been found to be more prone to premature myocardial infarction and adverse cardiovascular events than other groups [9]. In addition to conventional risk factors, psychosocial determinants have come to the fore as significant risk factors of CAD. Chronic stress and depression, as well as poor socioeconomic status, are increasingly being identified as independent risk factors affecting the development and progression of cardiovascular disease [10,11]. These are especially applicable in agrarian parts of India where financial instability, work-related stress, and agricultural-related uncertainty are major determinants of health.

Western Maharashtra, which has been linked to agrarian distress and poor health outcomes, is one of the regions that underscores the need to take into account these broader determinants in cardiovascular studies [12]. Although the burden of CAD in rural India is increasing, there is a lack of granular, region-specific epidemiological data. Extensive national and international research, including the CREATE registry and the INTERHEART study, has given useful information on cardiovascular risk factors and outcomes but does not give the localized information needed to comprehend local rural and peri-urban central India patterns [13,14]. Moreover, although registry-based and urban cohort studies have contributed to knowledge, hospital-based information from rural-serving tertiary care centers is scarce.

Here, the current cross-sectional study at the hospital was carried out in Krishna Hospital and Medical Research Centre to fill this knowledge gap. The objectives of the study were: (i) to fully describe the sociodemographic, lifestyle, psychosocial, and clinical characteristics of patients with CAD in rural and peri-urban central India; and (ii) to examine the relationships between major risk factors and the severity of the disease, especially multi-vessel disease (MVD), by bivariate and multivariate analyses.

## Materials and methods

Study design and setting

This hospital-based descriptive cross-sectional study was conducted in the Departments of General Medicine and Cardiology at Krishna Hospital & Medical Research Centre from June 2024 to May 2025. The study was designed and reported in accordance with the Strengthening the Reporting of Observational Studies in Epidemiology (STROBE) guidelines for cross-sectional studies [1]. Institutional Ethics Committee of Krishna Vishwa Vidyapeeth issued approval number KVV/IEC/08/2024. The study adhered to the ethical principles outlined in the Declaration of Helsinki [2]. Written informed consent was obtained from all participants before enrolment.

Study population and sample Size

Adult patients (≥18 years) with a confirmed diagnosis of coronary artery disease (CAD), including ST-elevation myocardial infarction (STEMI), non-ST-elevation myocardial infarction (NSTEMI), and unstable angina (UA), as defined by the Fourth Universal Definition of Myocardial Infarction [14], were included in the study. The sample size was calculated a priori using the standard formula for cross-sectional studies estimating a single proportion, $n = \frac{Z_{\alpha/2}^{2} \times p \times (1-p)}{d^{2}}$ where $n$ is the sample size, $Z_{\alpha/2}$​ is the standard normal deviate at the desired confidence level, p is the estimated prevalence, and d is the allowable error. Based on the prevalence of hypertension among Indian adults reported by Ramakrishnan et al. [9] (30%; p=0.30), with a 95% confidence level ($Z_{\alpha/2}​=1.96$) and an absolute precision of 9% ($d=0.09$), the estimated sample size was 99.6, which was rounded off to 100 participants. Accordingly, 100 consecutive eligible patients were enrolled. Patients aged ≥18 years with confirmed CAD were eligible for inclusion, while those with congenital heart disease, primary valvular heart disease, non-ischemic cardiomyopathies, or unwillingness to provide informed consent were excluded.

Data collection and variables

Data were collected using a structured and pre-tested case record form by trained personnel. Sociodemographic variables included age group, sex, educational level, marital status, employment status, household income, living arrangements, geographic location (rural or peri-urban), and religion. Anthropometric measurements included weight and body mass index (BMI), calculated as weight in kilograms divided by height in meters squared, and classified according to WHO Asia-Pacific criteria [15]. Lifestyle-related variables included smoking status, alcohol consumption, physical activity level (classified according to WHO 2020 guidelines), and dietary habits. Psychosocial parameters included stress levels assessed using a modified Perceived Stress Scale (PSS-4), depression and anxiety symptoms evaluated using PHQ-9 criteria, and assessment of the social support network. Clinical variables included diabetes status (defined as prior diagnosis or HbA1c ≥6.5%) [16, 17], hypertension (defined as prior diagnosis, current treatment, or blood pressure ≥140/90 mmHg on two or more readings) [16], and presenting complaints.

Laboratory parameters included HbA1c levels, lipid profile, cardiac biomarkers such as troponin-I and creatine phosphokinase-MB (CPK-MB), interpreted according to institutional reference standards. Electrocardiography (ECG) findings were interpreted by a consultant cardiologist and categorized as STEMI, T-wave inversion, other abnormalities, or normal. Echocardiographic assessment included measurement of left ventricular ejection fraction (EF), categorized as reduced (<50%) or preserved (≥50%), and the presence of regional wall motion abnormalities (RWMA). Coronary angiography (CAG), performed using the standard Judkins technique, was used to classify disease severity into single-vessel disease (SVD), double-vessel disease (DVD), or triple-vessel disease (TVD). The primary outcome variable was the presence of multi-vessel disease (MVD), defined as involvement of two or more coronary vessels (DVD and TVD), compared to SVD.

Statistical analysis

Data were entered into Microsoft Excel (Microsoft Corporation, Redmond, USA) and verified for accuracy before analysis. Statistical analysis was performed using IBM SPSS Statistics (IBM Corp., Armonk, NY, USA) version 27. As the dataset primarily comprised categorical variables, these were summarized using frequencies and percentages. Associations between study variables and the extent of coronary artery disease (single-vessel disease vs multi-vessel disease) were evaluated using the Chi-square test or Fisher’s exact test, as appropriate based on expected cell frequencies. Continuous variables were not a major component of the dataset and were therefore not subjected to parametric comparative testing. Multivariable binary logistic regression analysis was performed to identify independent predictors of multi-vessel disease.

Variables included in the model were selected based on clinical relevance and bivariable analysis findings and comprised age (>60 years), sex, living arrangement, marital status, depression/anxiety status, and smoking habit. The results were expressed as adjusted odds ratios with 95% confidence intervals, along with corresponding regression coefficients and Wald statistics. A two-tailed p-value of <0.05 was considered statistically significant.

## Results

A total of 100 patients with angiographically confirmed coronary artery disease (CAD) were included in the study. Among them, 39 (39.0%) had single-vessel disease (SVD), and 61 (61.0%) had multi-vessel disease (MVD), indicating a predominance of more extensive coronary involvement. The distribution of socio-demographic variables according to the extent of coronary artery disease (SVD vs MVD) is presented in Table 1.

**Table 1:** Association between socio-demographic characteristics and angiographic severity of coronary artery disease Percentages for SVD and MVD are calculated within each category unless otherwise specified; total sample size = 100; categories with zero frequency are retained for completeness and transparency; p-values were obtained using the Chi-square test or Fisher’s exact test, as appropriate; a p-value < 0.05 was considered statistically significant. n (%): number (percentage); SVD: Single-vessel disease; MVD: Multi-vessel disease; BMI: Body mass index; kg/m²: kilograms per square meter; χ2 value: Chi-square value; df: degree of freedom; p-value: asymptotic significance, 2-sided

Variable	Category	Total n (%)	SVD n (%)	MVD n (%)	χ2 value/df/p-value
Age group (years)	0–40	6 (6.0%)	4 (66.7%)	2 (33.3%)	χ2 = 7.279; p = 0.114 (Fisher Test)
	41–50	11 (11.0%)	6 (54.5%)	5 (45.5%)	
	51–60	26 (26.0%)	7 (26.9%)	19 (73.1%)	
	61–70	31 (31.0%)	15 (48.4%)	16 (51.6%)	
	>70	26 (26.0%)	7 (26.9%)	19 (73.1%)	
Sex	Male	61 (61.0%)	27 (44.3%)	34 (55.7%)	χ2 = 1.821; df = 1; p = 0.211
	Female	39 (39.0%)	12 (30.8%)	27 (69.2%)	
BMI (kg/m²)	<18.5	2 (2.0%)	0 (0.0%)	2 (100.0%)	χ2 = 1.769; p = 0.904 (Fisher Test)
	18.5–24.9	59 (59.0%)	23 (39.0%)	36 (61.0%)	
	25–29.9	31 (31.0%)	13 (41.9%)	18 (58.1%)	
	30–34.9	7 (7.0%)	3 (42.9%)	4 (57.1%)	
	35–39.9	1 (1.0%)	0 (0.0%)	1 (100.0%)	
	≥40	0 (0.0%)	0 (0.0%)	0 (0.0%)	
Education	No formal education	0 (0.0%)	0 (0.0%)	0 (0.0%)	χ2 = 2.281; p = 0.573 (Fisher Test)
	Up to Secondary education	46 (46.0%)	15 (32.6%)	31 (67.4%)	
	Higher secondary education	38 (38.0%)	18 (47.4%)	20 (52.6%)	
	Graduate	12 (12.0%)	5 (41.7%)	7 (58.3%)	
	Postgraduate	4 (4.0%)	1 (25.0%)	3 (75.0%)	
	Doctoral education	0 (0.0%)	0 (0.0%)	0 (0.0%)	
Marital status	Single	4 (4.0%)	3 (75.0%)	1 (25.0%)	χ2= 6.322; p = 0.057 (Fisher Test)
	Married	85 (85.0%)	35 (41.2%)	50 (58.8%)	
	Divorced	1 (1.0%)	0 (0.0%)	1 (100.0%)	
	Widowed	10 (10.0%)	1 (10.0%)	9 (90.0%)	
Employment status	Full-time	10 (10.0%)	5 (50.0%)	5 (50.0%)	χ2 = 3.831; p = 0.592 (Fisher Test)
	Unemployed	6 (6.0%)	3 (50.0%)	3 (50.0%)	
	Student	1 (1.0%)	1 (100.0%)	0 (0.0%)	
	Retired	29 (29.0%)	12 (41.4%)	17 (58.6%)	
	Homemaker	28 (28.0%)	8 (28.6%)	20 (71.4%)	
	Farmer	26 (26.0%)	10 (38.5%)	16 (61.5%)	
Income (rupees per month)	<10,000	12 (12.0%)	4 (33.3%)	8 (66.7%)	χ2 = 2.806; p = 0.610 (Fisher Test)
	10,000–50,000	51 (51.0%)	19 (37.3%)	32 (62.7%)	
	50,000–100,000	27 (27.0%)	13 (48.1%)	14 (51.9%)	
	100,000–200,000	7 (7.0%)	3 (42.9%)	4 (57.1%)	
	>200,000	3 (3.0%)	0 (0.0%)	3 (100.0%)	
Living arrangement	Alone	11 (11.0%)	7 (63.6%)	4 (36.4%)	χ2 = 5.484; p = 0.101 (Fisher Test)
	With Spouse	16 (16.0%)	4 (25.0%)	12 (75.0%)	
	With Family	72 (72.0%)	27 (37.5%)	45 (62.5%)	
	With Roommates	1 (1.0%)	1 (100.0%)	0 (0.0%)	
Location	Urban	17 (17.0%)	7 (41.2%)	10 (58.8%)	χ2 = 0.043; df = 2; p = 0.979
	Suburban	21 (21.0%)	8 (38.1%)	13 (61.9%)	
	Rural	62 (62.0%)	24 (38.7%)	38 (61.3%)	
Religion	Hindu	90 (90.0%)	35 (38.9%)	55 (61.1%)	χ2 = 0.005; p = 1.000 (Fisher Test)
	Muslim	10 (10.0%)	4 (40.0%)	6 (60.0%)	

Participants were predominantly in the older age groups, with the highest proportion in the 61-70 years category (31.0%), followed by 51-60 years (26.0%) and >70 years (26.0%). Across these strata, a higher proportion of MVD was observed in the older age groups; however, the association between age group and CAD extent was not statistically significant (p = 0.114). The study population demonstrated a male predominance, with 61.0% males and 39.0% females. Although MVD was more frequent among males compared to females, this difference did not reach statistical significance (p = 0.211). With respect to anthropometric status, the majority of participants were within the normal BMI range (18.5-24.9 kg/m²; 59.0%), followed by the overweight category (25-29.9 kg/m²; 31.0%), while fewer individuals were in higher BMI categories.

The distribution of SVD and MVD across BMI categories was comparable, with no statistically significant association (p = 0.904). Educational attainment was largely concentrated in the secondary (46.0%) and higher secondary (38.0%) levels, with smaller proportions of participants having graduate or postgraduate qualifications. The proportions of SVD and MVD across educational strata were similar, and no statistically significant association was observed (p = 0.573). In terms of marital status, the majority of participants were married (85.0%), followed by widowed (10.0%) and single (4.0%) individuals. A relatively higher proportion of MVD was observed among widowed participants; however, the overall association between marital status and CAD extent did not reach statistical significance (p = 0.057).

Employment status showed a heterogeneous distribution, with retired individuals (29.0%), homemakers (28.0%), and farmers (26.0%) comprising the largest groups. The distribution of CAD severity across employment categories was broadly similar, and no statistically significant association was identified (p = 0.592). Monthly household income was predominantly within the ₹10,000-₹50,000 range (51.0%), followed by ₹50,000-₹100,000 (27.0%), with smaller proportions in higher income categories. The proportions of SVD and MVD across income groups did not differ significantly (p = 0.610). Most participants reported living with family members (72.0%), followed by those living with a spouse (16.0%) and those living alone (11.0%). Although variation in the distribution of SVD and MVD was observed across these categories, the association was not statistically significant (p = 0.101). Geographically, a majority of participants were from rural areas (62.0%), with fewer from suburban (21.0%) and urban (17.0%) settings.

The distribution of CAD severity across geographic locations was comparable, with no statistically significant association (p = 0.979). Religious distribution indicated that 90.0% of participants were Hindu and 10.0% were Muslim. The proportions of SVD and MVD were similar across religious groups, and no statistically significant association was observed (p = 1.000).

The distribution of lifestyle and psychosocial factors in relation to the extent of coronary artery disease is presented in Table 2.

**Table 2:** Distribution of lifestyle and psychosocial characteristics according to the extent of coronary artery disease Categories with zero frequency are retained for completeness and transparency. P-values were obtained using the Chi-square test or Fisher’s exact test, as appropriate; a p-value < 0.05 was considered statistically significant. n (%): number (percentage); SVD: Single-vessel disease; MVD: Multi-vessel disease; χ2 value: Chi-square value; df: degree of freedom; p-value: asymptotic significance, 2-sided

Variable	Category	Total n (%)	SVD n (%)	MVD n (%)	χ2 value/df/p-value
Smoking Habit	Smoker	11 (11.0%)	7 (17.9%)	4 (6.6%)	χ2 = 3.153; p = 0.103 (Fisher Test)
	Non-smoker	89 (89.0%)	32 (82.1%)	57 (93.4%)	
Alcohol Consumption	Yes	21 (21.0%)	9 (23.1%)	12 (19.7%)	χ2 = 0.166; df = 1; p = 0.683
	No	79 (79.0%)	30 (76.9%)	49 (80.3%)	
Physical Activity Level	Sedentary	25 (25.0%)	10 (25.6%)	15 (24.6%)	χ2 = 0.484; df = 2; p = 0.785
	Light	59 (59.0%)	24 (61.5%)	35 (57.4%)	
	Vigorous	16 (16.0%)	5 (12.8%)	11 (18.0%)	
Dietary Habits	Vegetarian	19 (19.0%)	6 (15.4%)	13 (21.3%)	χ2 = 1.832; p = 0.416 (Fisher Test)
	Non-vegetarian	9 (9.0%)	2 (5.1%)	7 (11.5%)	
	Mixed	72 (72.0%)	31 (79.5%)	41 (67.2%)	
Stress Levels	Low	38 (38.0%)	16 (41.0%)	22 (36.1%)	χ2 = 1.651; p = 0.473 (Fisher Test)
	Moderate	55 (55.0%)	19 (48.7%)	36 (59.0%)	
	High	7 (7.0%)	4 (10.3%)	3 (4.9%)	
Depression Levels	None	57 (57.0%)	17 (43.6%)	40 (65.6%)	χ2 = 5.832; p = 0.081 (Fisher Test)
	Mild	33 (33.0%)	16 (41.0%)	17 (27.9%)	
	Moderate	9 (9.0%)	5 (12.8%)	4 (6.6%)	
	Severe	1 (1.0%)	1 (2.6%)	0 (0.0%)	
Social Support Network	Family	72 (72.0%)	29 (74.4%)	43 (70.5%)	χ2 = 0.691; p = 0.758 (Fisher Test)
	Friends	17 (17.0%)	7 (17.9%)	10 (16.4%)	
	Family + Friends	11 (11.0%)	3 (7.7%)	8 (13.1%)	
	Support group	0 (0.0%)	0 (0.0%)	0 (0.0%)	

With respect to smoking status, the majority of participants were non-smokers (89.0%), while 11.0% were smokers. A higher proportion of SVD was observed among smokers compared to MVD, whereas non-smokers demonstrated a greater proportion of MVD. However, this difference was not statistically significant (p = 0.103). In terms of alcohol consumption, 21.0% of participants reported alcohol use, while 79.0% did not. The distribution of SVD and MVD across these categories was comparable, with no statistically significant association observed (p = 0.683). Physical activity levels were predominantly light (59.0%), followed by sedentary (25.0%) and vigorous (16.0%) activity. The proportion of SVD and MVD across these categories remained similar, and no statistically significant association was identified (p = 0.785).

Dietary patterns indicated that the majority of participants followed a mixed diet (72.0%), with smaller proportions adhering to vegetarian (19.0%) and non-vegetarian (9.0%) diets. The distribution of CAD severity across dietary categories did not differ significantly (p = 0.416). Stress levels were most commonly reported as moderate (55.0%), followed by low (38.0%) and high (7.0%) levels. The proportions of SVD and MVD were comparable across these categories, with no statistically significant association (p = 0.473).

Assessment of depression levels showed that 57.0% of participants had no depression, while 33.0% had mild depression, and smaller proportions had moderate or severe depression. A higher proportion of MVD was observed among individuals without depression, whereas relatively higher proportions of SVD were noted among those with mild and moderate depression. However, this distribution did not reach statistical significance, although it approached significance (p = 0.081). With regard to social support, the majority of participants reported family-based support (72.0%), followed by support from friends (17.0%) and combined family and friends (11.0%). No participants reported reliance on support groups. The distribution of SVD and MVD across social support categories was similar, and no statistically significant association was observed (p = 0.758).

Table 3 presents the comparative distribution of clinical comorbidities according to the extent of coronary artery disease (CAD).

**Table 3:** Comparative distribution of diabetes mellitus and hypertension across single and multi vessel coronary artery disease P-values were obtained using the Chi-square test or Fisher’s exact test, as appropriate; a p-value < 0.05 was considered statistically significant. n (%): number (percentage); SVD: single vessel disease; MVD: multi vessel disease; CAD: coronary artery disease; χ2 value: Chi-square value; df: degree of freedom; p-value: asymptotic significance, 2-sided

Variable	Category	Total n (%)	SVD n (%)	MVD n (%)	χ2 value/df/p-value
Diabetes Mellitus	Yes	37 (37%)	15 (40.5%)	22 (59.5%)	χ2 = 0.059; df = 1; p = 0.809
	No	63 (63%)	24 (38.1%)	39 (61.9%)	
Hypertension	Yes	59 (59%)	22 (37.3%)	37 (62.7%)	χ2 = 0.177; df = 1; p = 0.674
	No	41 (41%)	17 (41.5%)	24 (58.5%)	

Diabetes mellitus was identified in 37.0% of participants, whereas 63.0% were non-diabetic. Among individuals with diabetes, 59.5% had multi-vessel disease (MVD) and 40.5% had single-vessel disease (SVD). A similar distribution was observed among non-diabetic participants, with 61.9% having MVD and 38.1% having SVD. The association between diabetes status and the extent of CAD was not statistically significant (p = 0.809). Hypertension was present in 59.0% of participants, while 41.0% did not have hypertension. Among hypertensive individuals, 62.7% had MVD compared to 58.5% among non-hypertensive individuals. The proportion of SVD was 37.3% in hypertensive participants and 41.5% in those without hypertension. No statistically significant association was observed between hypertension status and CAD extent (p = 0.674).

The distribution of electrocardiographic and biochemical parameters according to the extent of coronary artery disease is presented in Table 4.

**Table 4:** Distribution of electrocardiographic and biochemical parameters according to the extent of coronary artery disease P-value < 0.05 was considered statistically significant. n (%): number (percentage); SVD: Single-vessel disease; MVD: Multi-vessel disease; ECG: Electrocardiogram; STEMI: ST-elevation myocardial infarction; EF: Ejection fraction; Trop-I: Troponin-I; CPK-MB: Creatine phosphokinase-MB; CAD: Coronary artery disease; χ2 value: Chi-square value; df: degree of freedom; p-value: asymptotic significance, 2-sided

Variable	Category	Total n (%)	SVD n (%)	MVD n (%)	χ2 value/df/p-value
ECG Findings	ST-segment elevation (STEMI pattern)	40 (40.0%)	16 (41.0%)	24 (39.3%)	χ2 = 1.549; df = 3; p = 0.671
	ST-segment depression and/or T-wave inversion (ischemic changes)	28 (28.0%)	9 (23.1%)	19 (31.1%)	
	Other ECG abnormalities (e.g., arrhythmias, bundle branch block, nonspecific changes)	18 (18.0%)	9 (23.1%)	9 (14.8%)	
	Normal ECG	14 (14.0%)	5 (12.8%)	9 (14.8%)	
Ejection Fraction (EF)	Reduced	39 (39.0%)	17 (43.6%)	22 (36.1%)	χ2 = 0.566; df = 1; p = 0.452
	Preserved	61 (61.0%)	22 (56.4%)	39 (63.9%)	
Troponin-I (Trop-I)	Positive	80 (80.0%)	31 (79.5%)	49 (80.3%)	χ2 = 0.011; df = 1; p = 0.918
	Negative	20 (20.0%)	8 (20.5%)	12 (19.7%)	
CPK-MB	Positive	76 (76.0%)	30 (76.9%)	46 (75.4%)	χ2 = 0.030; df = 1; p = 0.863
	Negative	24 (24.0%)	9 (23.1%)	15 (24.6%)	
Lipid Profile	Deranged	80 (80.0%)	34 (87.2%)	46 (75.4%)	χ2 = 2.060; df = 1; p = 0.151
	Normal	20 (20.0%)	5 (12.8%)	15 (24.6%)	

Electrocardiographic (ECG) findings demonstrated that ST-segment elevation (STEMI pattern) was the most common presentation (40.0%), followed by ischemic changes (28.0%), other ECG abnormalities (18.0%), and normal ECG findings (14.0%). The proportion of SVD and MVD across these ECG categories was comparable, and no statistically significant association was observed between ECG findings and CAD extent (p = 0.671). Assessment of left ventricular function revealed that 39.0% of participants had reduced ejection fraction, while 61.0% had preserved ejection fraction. Although a higher proportion of reduced ejection fraction was observed in the SVD group compared to the MVD group, this difference was not statistically significant (p = 0.452).

Biochemical markers showed that troponin-I was positive in 80.0% of participants, with a similar distribution between SVD and MVD groups. No statistically significant association was identified between troponin-I status and CAD extent (p = 0.918). Similarly, CPK-MB positivity was observed in 76.0% of participants, with comparable proportions in SVD and MVD groups. The association between CPK-MB levels and CAD severity was not statistically significant (p = 0.863). Lipid profile assessment indicated that 80.0% of participants had a deranged lipid profile, while 20.0% had normal lipid levels. The distribution of CAD extent across lipid profile categories did not differ significantly (p = 0.151). Overall, electrocardiographic patterns, left ventricular function, and biochemical markers demonstrated similar distributions between SVD and MVD groups, with no statistically significant associations observed.

Multivariate logistic regression analysis was performed to identify independent predictors of multi-vessel coronary artery disease (MVD) as presented in Table 5.

**Table 5:** Multivariate logistic regression analysis for predictors of multi-vessel coronary artery disease *Statistically significant (p < 0.05) B: regression coefficient; Exp(B): exponentiated coefficient (adjusted odds ratio); CI: confidence interval; MVD: Multi-vessel disease; SVD: Single-vessel disease; p-value: asymptotic significance, 2-sided;

Study Variable	Regression Coefficient (B)	Wald Statistic	P-value	Adjusted Odds Ratio (Exp(B))	95% CI Lower	95% CI Upper
Age (>60 years)	-0.191	0.174	0.676	0.826	0.337	2.024
Sex (Male)	-0.329	0.427	0.513	0.72	0.269	1.928
Living arrangement (Alone/others)	-2.037	5.098	0.024*	0.13	0.022	0.764
Marital status (Not married)	1.469	2.489	0.115	4.343	0.701	26.92
Depression/Anxiety (Present)	-0.574	1.602	0.206	0.563	0.232	1.37
Smoking habit (Smoker)	-1.122	2.069	0.15	0.325	0.071	1.502

After adjustment for potential confounders, living arrangement emerged as the only statistically significant predictor of CAD extent. Participants categorized under “alone/other living arrangements” had significantly lower odds of MVD compared to those living with family or spouse (adjusted OR = 0.13; 95% CI: 0.02-0.76; p = 0.024). Other variables, including age group, sex, marital status, depression/anxiety status, and smoking habit, were not significantly associated with the extent of CAD after adjustment (p > 0.05). Although marital status demonstrated increased odds of MVD among non-married individuals (adjusted OR = 4.34), this association did not reach statistical significance (p = 0.115). Similarly, smoking habit and depression/anxiety status showed reduced odds of MVD; however, these associations were not statistically significant. Age and sex also did not demonstrate a significant relationship with CAD severity in the adjusted model.

## Discussion

The present cross-sectional study examined the sociodemographic, lifestyle, psychosocial, and clinical determinants of angiographic severity of coronary artery disease (CAD) among 100 patients from Western Maharashtra with angiographically confirmed disease. Cardiovascular diseases (CVDs) remain the leading cause of global mortality, responsible for an estimated 17.9 million deaths annually [3,4]. In India, CAD has emerged as a particularly significant public health burden, with epidemiological data documenting a rising incidence, an earlier age of onset, and a distinctly adverse risk factor profile compared to Western populations [5,6,7]. Urban Indian population data have highlighted a growing prevalence of multiple conventional cardiovascular risk factors, including hypertension, diabetes, and dyslipidemia, that collectively accelerate coronary atherosclerosis [8].

The national prevalence of hypertension among Indian adults has been estimated at approximately 29.5% in community-based surveys [9], while psychosocial stressors have been independently linked to cardiovascular disease development and progression [10]. In agrarian communities of Maharashtra in particular, chronic psychological morbidity among farming populations adds a dimension of occupational psychosocial risk to the already high-burden cardiovascular milieu [11]. Against this backdrop, the present study identified a predominance of multi-vessel disease (MVD), with 61.0% of participants exhibiting involvement of more than one coronary vessel and 39.0% having single-vessel disease (SVD). This distribution aligns with patterns documented in large Indian acute coronary syndrome (ACS) registries [12] and reflects the established propensity of South Asian populations for diffuse and premature coronary atherosclerosis, as demonstrated in the INTERHEART study [13]. The diagnosis and classification of myocardial infarction in the present population were guided by the Fourth Universal Definition of Myocardial Infarction [14].

To ensure methodological rigor, anthropometric classification was performed using Asian-specific BMI thresholds, which recommend lower cut-off values for overweight and obesity in South Asian populations given their higher visceral adiposity at comparatively lower BMI levels [15]. Hypertension was defined in accordance with the 2018 European Society of Cardiology (ESC) and the European Society of Hypertension (ESH) (ESC/ESH) Guidelines for the management of arterial hypertension [16], and diabetes mellitus was diagnosed and classified based on the American Diabetes Association Standards of Care 2023 [17]. Psychosocial assessment employed the Perceived Stress Scale (PSS) for quantification of perceived stress [18] and the Patient Health Questionnaire-9 (PHQ-9) for grading of depression severity [19]. Physical activity levels were categorized in accordance with the World Health Organization 2020 guidelines on physical activity and sedentary behaviour [20].

Atherosclerosis is a complex and progressive inflammatory disease involving lipid accumulation, endothelial dysfunction, and vascular inflammation, contributing to the development and progression of coronary artery disease [21]. Echocardiographic assessment of left ventricular function was performed following the recommendations of the American Society of Echocardiography and the European Association of Cardiovascular Imaging [22]. Coronary angiography was performed and interpreted according to American College of Cardiology/American Heart Association (ACC/AHA) guidelines for coronary angiography [23], with troponin-I positivity determined using the 99th percentile upper reference limit for the assay in use [24]. The patient profile in this study is broadly consistent with that of previously reported Indian ACS registries, including the Kerala ACS Registry, which documented the clinical and sociodemographic characteristics of hospitalized ACS patients across South India [25].

Sociodemographic characteristics and CAD severity

The study demonstrated a male predominance (61.0%), with MVD being proportionally more common among females (69.2%) than males (55.7%), though this difference was not statistically significant (p = 0.211). The higher relative MVD burden in women is consistent with established literature: women tend to present with more advanced coronary disease at the time of diagnosis, partly attributable to referral bias and differential symptom recognition patterns previously documented in studies on sex-based disparities in CAD management [26,27]. Hormonal protection conferred by estrogen before menopause delays clinical CAD manifestation, resulting in older age at presentation and, frequently, more diffuse atherosclerotic involvement at the time of angiographic assessment.

Educational attainment, employment status, monthly household income, living arrangement, geographic location, marital status, and religion were not significantly associated with CAD extent (all p > 0.05). In the Indian context, socioeconomic factors, including income level and educational status, have been shown to influence cardiovascular risk through differential access to healthcare and health-promoting resources, as demonstrated in studies on the socioeconomic burden of non-communicable diseases in below-poverty-line households in South India [28]. The predominance of rural participants (62.0%) in this population is particularly noteworthy; rural populations in Maharashtra may face barriers to timely cardiac care, potentially contributing to delayed presentation and more extensive coronary disease at the time of angiographic diagnosis. The distribution of SVD and MVD across BMI categories (predominantly normal range, 18.5-24.9 kg/m²; 59.0%) was comparable, with no significant association (p = 0.904), consistent with the recognized limitation of BMI as an adiposity surrogate in South Asians [15].

Participants were predominantly in the older age strata, with the 61-70 years age group constituting the largest proportion (31.0%), followed by those aged 51-60 years and above 70 years (26.0% each). A trend toward greater MVD prevalence was observed with advancing age, particularly in the 51-60 years (73.1% MVD) and above 70 years (73.1% MVD) groups, though this association did not achieve statistical significance (p = 0.114). Prolonged exposure to cardiovascular risk factors with advancing age, including type 2 diabetes, which carries a substantially elevated coronary mortality risk in affected individuals [29], fosters progressive and diffuse coronary atherosclerosis. The UKPDS 35 study demonstrated that each 1% reduction in HbA1c was associated with a meaningful reduction in macrovascular complications [30], suggesting that the duration and glycemic control of diabetes may influence the anatomical extent of coronary involvement.

Notably, South Asian populations are known to develop CAD at a younger age than their Western counterparts. Data from the Tamil Nadu STEMI program demonstrated that a significant proportion of STEMI presentations in India occur among individuals below 45 years of age [31], while Joshi et al. confirmed that South Asians develop myocardial infarction at a younger age than populations in other countries, with an adverse risk factor burden apparent earlier in life [32]. The absence of a statistically significant age-severity association in the present study may reflect the modest sample size and the cross-sectional design.

Lifestyle factors and psychosocial determinants

Diabetes mellitus was identified in 37.0% of participants, with comparable MVD proportions among diabetic (59.5%) and non-diabetic (61.9%) individuals (p = 0.809). While diabetes is well-established as a major accelerator of coronary atherosclerosis [29], the absence of a significant association in the present study may reflect insufficient statistical power, the cross-sectional design, and heterogeneity in glycemic control status among participants. The diabetes prevalence in this population (37.0%) is consistent with reported rates in South Indian peri-urban populations from the Chennai Urban Rural Epidemiology Study (CURES-17) [33], reflecting the high metabolic risk burden characterizing Indian communities.

Hypertension was present in 59.0% of participants. Among hypertensive individuals, 62.7% had MVD compared to 58.5% of non-hypertensive participants (p = 0.674). The elevated hypertension prevalence in this hospitalized population [9] may reflect referral of a high-risk patient population, and the non-significant association with CAD severity may be partly confounded by ongoing antihypertensive therapy. Lipid profile was deranged in 80.0% of participants, with a numerically higher abnormality rate in the SVD group (87.2% vs. 75.4%), though without statistical significance (p = 0.151). Dyslipidemia is among the most potent modifiable risk factors for atherosclerotic cardiovascular disease [13], and its high prevalence in this population is consistent with the European Society of Cardiology and European Atherosclerosis Society (ESC/EAS) 2020 Guidelines on dyslipidemia management, which emphasize low-density lipoprotein (LDL)-cholesterol reduction as central to reducing both primary and recurrent cardiovascular events [34]. The absence of a differential lipid burden between SVD and MVD groups suggests that dyslipidemia may function as a threshold risk factor enabling the initiation of coronary atherosclerosis, with angiographic severity subsequently shaped by additional modifiers such as disease duration and genetic susceptibility.

Depression, assessed using the PHQ-9 [19], was present in 43.0% of participants 33.0% mild, 9.0% moderate, and 1.0% severe. A trend approaching statistical significance was observed (p = 0.081), with individuals without depression showing a higher proportion of MVD (65.6%), while those with mild or moderate depression demonstrated relatively greater SVD rates. This clinically meaningful finding is consistent with evidence that depression serves as an independent risk factor for poor prognosis among patients with acute coronary syndrome [35]. The higher depression burden in the SVD group may reflect the acute psychological impact of a major cardiac diagnosis, irrespective of anatomical disease extent. This near-significant association warrants prospective investigation to clarify directionality. Perceived stress levels, assessed using the PSS [18], were predominantly moderate (55.0%), with no statistically significant association with CAD extent (p = 0.473). While psychosocial stress promotes cardiovascular disease through neuroendocrine and inflammatory pathways [10], cross-sectional data limit causal inference.

With respect to smoking status, 11.0% of participants were smokers. Counterintuitively, a higher proportion of SVD was observed among smokers compared to MVD (p = 0.103, non-significant). This may be partly explained by the widespread use of smokeless tobacco in rural Maharashtra, which is frequently underreported in standard smoking surveys. Smokeless tobacco use among South Asians carries substantial cardiovascular risk through nicotine-mediated platelet aggregation and endothelial dysfunction, as documented by Siddiqi et al. [36], and its absence from formal documentation in this study may have attenuated the observed relationship between tobacco exposure and angiographic severity. Physical activity levels were predominantly light (59.0%), followed by sedentary (25.0%) and vigorous (16.0%), with no significant association with CAD severity (p = 0.785). While physical inactivity is a well-recognized modifiable cardiovascular risk factor [20], the high proportion of farming households (26.0%) in this population, whose occupational physical demands may not be captured by standard activity instruments, may have diluted this association. Dietary patterns and alcohol consumption were similarly not significantly associated with CAD extent.

Electrocardiographic and biochemical parameters

ST-elevation myocardial infarction-pattern ECG changes were the most prevalent electrocardiographic finding (40.0%), followed by ischemic changes (28.0%), other ECG abnormalities (18.0%), and normal ECG (14.0%). The distribution of ECG patterns was broadly comparable across SVD and MVD groups (p = 0.671). This is consistent with the recognized limitation of the ECG in discriminating anatomical coronary burden: it primarily reflects culprit-territory ischemia rather than total coronary involvement, a concept fundamental to both the Third [21] and Fourth Universal Definitions of Myocardial Infarction [14]. Coronary angiography was performed according to ACC/AHA guidelines [23], ensuring standardized assessment of disease extent. Left ventricular ejection fraction (LVEF), assessed by echocardiography per American Society of Echocardiography and the European Association of Cardiovascular Imaging (ASE/EACVI) recommendations [22], was preserved in 61.0% and reduced in 39.0% of participants.

Although reduced LVEF was numerically more prevalent in the SVD group (43.6% vs. 36.1%), this difference was not statistically significant (p = 0.452), possibly because large-territory SVD infarcts, particularly in the left anterior descending artery distribution, can cause substantial LV dysfunction despite single-vessel involvement. Troponin-I was positive in 80.0% of participants [24], with similar distributions across SVD and MVD groups (p = 0.918), while CPK-MB positivity was observed in 76.0% (p = 0.863), reflecting the predominantly acute and sub-acute clinical presentations in this hospitalized population.

Multivariate predictors of multi-vessel disease

Multivariate logistic regression analysis, adjusting for age, sex, marital status, depression/anxiety status, and smoking habit, identified living arrangement as the sole independent predictor of MVD in this population. Participants in alone or other non-family living arrangements demonstrated significantly lower odds of MVD compared to those residing with family or a spouse (adjusted OR = 0.13; 95% CI: 0.02-0.76; p = 0.024). This finding is novel and requires careful interpretation. Individuals living with family in this rural-predominant population may be more exposed to shared household dietary patterns, reduced individual mobility, and a greater cumulative cardiovascular risk factor burden accumulated over decades of shared lifestyle, thereby promoting more extensive coronary atherosclerosis over time. Conversely, individuals living alone may represent a younger, more mobile, or occupationally active subgroup with a different risk trajectory.

An alternative explanation relates to healthcare access: those living without family support may seek medical attention earlier due to the absence of household-based caregiving, resulting in earlier diagnosis and relatively less advanced coronary disease at angiography. The complex and bidirectional relationship between social support and cardiovascular risk is well-recognized [10]; in some contexts, the household environment itself may serve as a conduit for shared lifestyle risk rather than a protective buffer. This association should be interpreted cautiously, given the small number of participants living alone (n = 11), which limits the stability of the regression estimate and accounts for the wide confidence intervals (95% CI: 0.02-0.76). Replication in larger, population-representative prospective populations is warranted.

Marital status (not married) demonstrated a numerically elevated adjusted odds of MVD (OR = 4.34; p = 0.115), consistent with epidemiological evidence linking widowhood and spousal bereavement with adverse cardiovascular outcomes through chronic psychosocial stress pathways [10]. Smoking habit, depression/anxiety status, age, and sex were not significant independent predictors of MVD after multivariate adjustment, likely reflecting the multifactorial and interrelated nature of coronary atherosclerotic burden and the limited sample size.

Strengths and limitations

This study has several notable strengths. It adhered to STROBE reporting guidelines for observational research [1] and the ethical principles of the Declaration of Helsinki [2]. All CAD diagnoses were objectively confirmed by coronary angiography using established ACC/AHA criteria [23]. Validated instruments were employed for assessment of key variables, including the Perceived Stress Scale (PSS) for stress [18], the PHQ-9 for depression [19], and the WHO 2020 criteria for physical activity [20]. Comorbidities were defined using internationally recognized criteria [16,17], echocardiographic evaluation followed ASE/EACVI recommendations [22], and troponin-I positivity was determined using the 99th percentile upper reference limit [24]. These methodological strengths enhance the internal validity and clinical relevance of the findings. However, certain limitations should be acknowledged.

The cross-sectional design limits causal inference, and the relatively small sample size (n=100) may reduce statistical power, particularly for multivariable analyses. As a single-center, hospital-based study from a tertiary care setting in Western Maharashtra, the findings may be subject to selection bias and limited generalizability. Additionally, important variables such as smokeless tobacco use, duration and control of diabetes, family history of premature CAD, renal function, inflammatory markers (e.g., high-sensitivity C-reactive protein (hs-CRP)), pharmacological therapy, and genetic predisposition were not assessed.

Despite these limitations, the study provides meaningful region-specific insights. Future large-scale, prospective, community-based studies incorporating comprehensive clinical, biochemical, and genetic profiling are warranted to better understand the determinants of multi-vessel CAD in this population.

## Conclusions

In this hospital-based cohort from Western Maharashtra, multi-vessel coronary artery disease (MVD) was the predominant angiographic phenotype, indicating a high burden of advanced atherosclerotic disease at presentation and mirroring the contemporary epidemiology of CAD in India. Despite a high prevalence of established risk factors like hypertension, dyslipidaemia, and diabetes, no individual factor independently predicted angiographic severity, underscoring the complex, multifactorial nature of disease progression and the limitations of conventional risk stratification in this setting. Living arrangement emerged as the sole independent correlate of MVD, with individuals living alone demonstrating lower odds of multi-vessel involvement, highlighting a potentially under-recognised role of social context in shaping disease expression.

Signals observed with advancing age, widowhood, and depressive symptoms further support the relevance of psychosocial determinants in cardiovascular pathology. Taken together, these findings argue for a shift beyond traditional biomedical models toward integrated, biopsychosocial frameworks for risk assessment and prevention. Scaled, prospective studies incorporating granular clinical, behavioural, and genetic data are needed to refine risk prediction and inform context-specific strategies to reduce the burden of advanced CAD in underserved populations.
